# Offline Memory Reprocessing: Involvement of the Brain's Default Network in Spontaneous Thought Processes

**DOI:** 10.1371/journal.pone.0004867

**Published:** 2009-03-17

**Authors:** Kun Wang, Chunshui Yu, Lijuan Xu, Wen Qin, Kuncheng Li, Lin Xu, Tianzi Jiang

**Affiliations:** 1 National Laboratory of Pattern Recognition, Institute of Automation, Chinese Academy of Sciences, Beijing, People's Republic of China; 2 Department of Radiology, Xuanwu Hospital of Capital Medical University, Beijing, People's Republic of China; 3 Laboratory of Learning and Memory, Kunming Institute of Zoology, Chinese Academy of Sciences, Kunming, People's Republic of China; Indiana University, United States of America

## Abstract

**Background:**

Spontaneous thought processes (STPs), also called daydreaming or mind-wandering, occur ubiquitously in daily life. However, the functional significance of STPs remains largely unknown.

**Methodology/Principal Finding:**

Using functional magnetic resonance imaging (fMRI), we first identified an STPs-network whose activity was positively correlated with the subjects' tendency of having STPs during a task-free state. The STPs-network was then found to be strongly associated with the default network, which has previously been established as being active during the task-free state. Interestingly, we found that offline reprocessing of previously memorized information further increased the activity of the STPs-network regions, although during a state with less STPs. In addition, we found that the STPs-network kept a dynamic balance between functional integration and functional separation among its component regions to execute offline memory reprocessing in STPs.

**Conclusion/Significance:**

These findings strengthen a view that offline memory reprocessing and STPs share the brain's default network, and thus implicate that offline memory reprocessing may be a predetermined function of STPs. This supports the perspective that memory can be consolidated and modified during STPs, and thus gives rise to a dynamic behavior dependent on both previous external and internal experiences.

## Introduction

An active internal mental life is a common human experience. Neural imaging studies have found that the human brain is highly active even when it is not engaged in specific tasks [1]. Spontaneous thought processes (STPs), also referred to as daydreaming [2], [3] or mind-wandering [4], occur frequently in the absence of, or along with low levels of, demand from a task [5]. Since STPs are ubiquitous in everyday life, researchers have suggested that they constitute a psychological baseline for human brain functions [6], [7]. Despite their prevalence, the functional significance of STPs remains largely unknown. Some studies have suggested that STPs enable individuals to maintain an optimal level of arousal [6], [8]. However, the contents of STPs include everything from mundane recounts of recent happenings to plans and expectations about the future [4], [9]–[12], causing us to hypothesize a more complex role with additional functions to that mentioned above.

In the present study, we hypothesized that offline memory reprocessing is an important underlying process of STPs. Offline memory reprocessing has been used to refer to the process during which the brain cuts out normal input from the outside world, and looks for older memories that are relevant to memories from the recent past to see if the older memories can be usefully linked to the newer ones [13]–[15]. Through offline memory reprocessing, the brain accomplishes memory consolidation [13]–[15] and memory revision [16], [17]. Depending on daily experiences, daydreaming/mind-wandering usually occurs in the context of memory [16], [17]. In addition, although the memorization of events or facts is believed to mainly involve the hippocampus and associated cortices [18], [19], recent studies have found that offline memory reprocessing usually activates the precuneus and some ventrolateral and dorsolateral prefrontal regions [20]–[22]. These regions belong to an active default network during the task-free state [1], [8], [23], in which STPs are most likely to occur [4], [6], [7]. Therefore, we suspect that offline memory reprocessing, which is thought to mainly take place during sleep [13]–[15], may also happen automatically during STPs.

To test this hypothesis, we used spontaneous activity and functional connectivity MRI to explore the relationship between offline memory reprocessing and STPs. In this study, participants were first instructed to maintain a natural resting state (N-Rest) during a six-minute fMRI scan by closing their eyes, relaxing their minds and stopping any structured mental processes such as counting. About one hour after the N-Rest scans, those subjects were asked to memorize details of a picture for about ten minutes, and a second scan was performed after the memory task. During the second six-minute scan, the subjects were requested to maintain a state by closing their eyes and recalling the details of the picture. For each subject, we did a thought query immediately after the scan. According to their reports, they were mainly involved in the following cognitive processes: (1) recalling the details of the picture (contents, color, shape and so on); (2) picture-related imagination; (3) picture-related processing: they reported that the retrieved picture contents seems to change during the scanning period; (4) picture-unrelated STPs. The subjects spent most of time (85±10%, mean±SD, data can be seen in Table S1 of Supplementary Materials) on the offline reprocessing of memorized information (included all those picture-related processes). Therefore, we named the second state as offline memory reprocessing (M-Reprocess) state. We first identified the neural network associated with STPs (“STPs-network”), and then investigated the modulations of activities and interactions within the STPs-network by offline memory reprocessing. The results highlight the relationship between STPs and offline memory reprocessing, and offer convergent evidence for our hypothesis that offline memory reprocessing is a possible additional function of STPs.

## Results

In this study, we used regional homogeneity (ReHo) to measure the spontaneous activity level [24]. The ReHo method is based on the assumption that the activities of voxels within a functional brain area are more temporally synchronous in a high state of activity than in a low state. In the paper in which we described the ReHo methodology [24], we demonstrated that motor-related brain regions showed a higher ReHo value during a motor task than in the resting state. Additionally, the posterior cingulate cortex, a critical node in the default mode network [23], [25], showed a higher ReHo value in the resting state than in the task state. These findings suggest that ReHo values, although not directly measuring neuronal activity, can be used to assess brain activity level.

**Figure 1:**
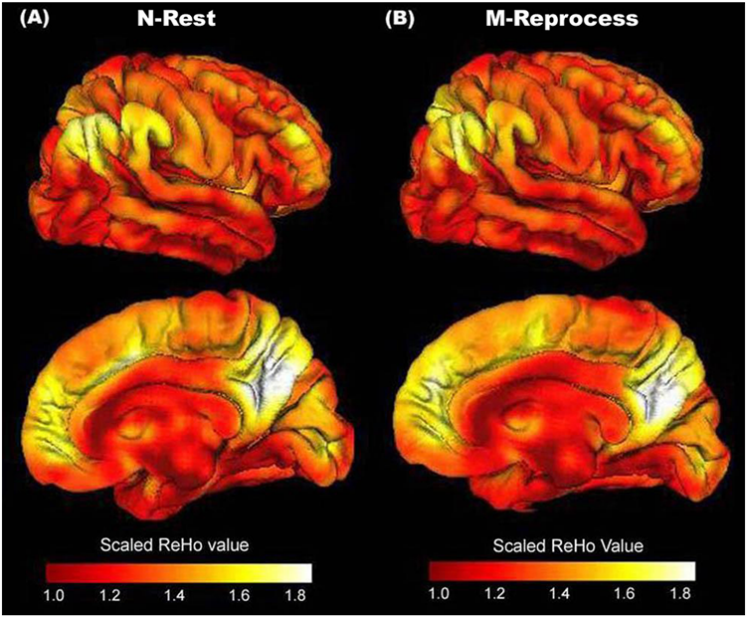
Mean ReHo-reflected activity maps during the N-Rest (A) and the M-Reprocess (B). The mean within-group maps were merely used for visualizing the ReHo-reflected activity. Visual inspection indicates that the default network showed high ReHo-reflected activity during both the N-Rest and the M-Reprocess in the posterior cingulate cortex, precuneus, bilateral inferior parietal cortex, the medial prefrontal cortex, and bilateral dorsal lateral prefrontal cortex.

As shown in Figure 1, visual inspection indicated a high ReHo-reflected activity in the posterior cingulate cortex, the precuneus, the medial prefrontal cortex, bilateral inferior parietal cortex and bilateral dorsal lateral prefrontal cortex during the N-Rest period. Such a network is consistent with the brain's default network, which has been found to be active during the resting state [23], [25]. This result further indicates that the ReHo value can be used as an index of brain activity level. Moreover, the activity pattern during the M-Reprocess appears similar to that of the N-Rest, possibly suggesting that offline memory reprocessing, the dominant process during the M-Reprocess, is also an important process during the N-Rest.

**Figure 2:**
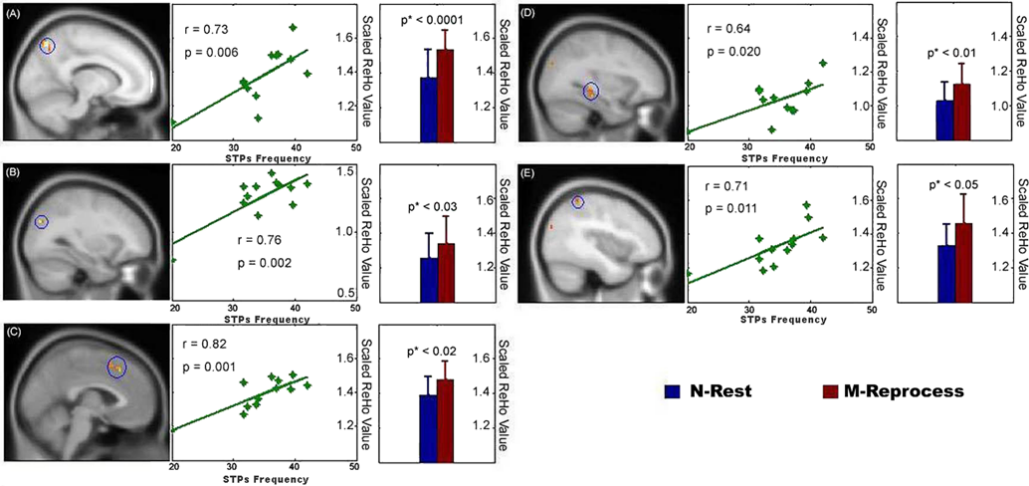
The STPs-network Regions. The ReHo-reflected activity in those regions were significantly correlated with subjects' STPs frequency scales (total score = 60) during the N-Rest, and were significantly stronger during the M-Reprocess than during the N-Rest. (A) PCu (BA 7; (−12, −69, 51); cluster = 729 mm^3^). (B) AG/SOG (BA 39/19; (−30, −78, 30); (−42, −81, 24); cluster = 270 mm^3^). (C) mPFG (BA 8/6; (−3, 24, 45); (−3, 15, 51); cluster = 297 mm^3^). (D) HIP/PHIP ((−31, −38, −13); (−33, −33, −3); cluster = 324 mm^3^). (E) IPL (BA 40; (−39, −48, 57); cluster = 243 mm^3^). All coordinates were in the Montreal Neurological Institute (MNI) space. The p* value is a corrected significance determined by Monte Carlo simulation.

In the present study, we quantified the subjects' tendencies to have STPs by measuring their daydreaming frequencies using the Imaginal Process Inventory (IPI) [26] (details of their scores can be seen in Table S1 and Figure 2). It should be noted that we can not measure the precise amount of STPs during the scanning. However, it has been suggested that individuals exhibit individually consistent differences in their propensity to daydream/mind-wander [3]. Therefore, the IPI-measured STPs frequencies could be used to evaluate the differences across subjects. We speculated that if a subject tends to have more STPs, the STPs-network regions would have a stronger activity during the N-Rest. Therefore, we first performed a voxel-wise correlation analysis between individuals' STPs frequencies and their ReHo-reflected activities during N-Rest. We then converted the resultant network (with a threshold of *P*<0.05 for individual voxels and cluster size >5 voxels; network details can be seen in Figure S1 of Supplementary materials) into a binary image and used it as an “inclusive” mask in the subsequent analysis. Next, to test whether the activity of the STPs-network regions could be modulated by externally evoked offline memory reprocessing, a random-effect paired-*t* test was performed between the M-Reprocess and the N-Rest (with a combined threshold of *P*<0.05 for individual voxels and a cluster size >216 mm^3^, equal to 8 voxels. This yield a corrected threshold of *P*<0.05 for multiple comparisons within the exclusive mask, as determined by a Monte Carlo simulation using the AFNI AlphaSim program [27]).

Our results indicated that the STPs-network regions, including the left precuneus (PCu, BA 7), the left angular gyrus/superior occipital gyrus (AG/SOG, BA 39/19), the left inferior parietal lobule (IPL, BA 40), the medial prefrontal gyrus (mPFG, BA 8/6) and the left hippocampus/parahippocampus (HIP/PHIP), showed significant correlations with the subjects' mind-wandering frequencies (Figure 2). These STPs-network regions also showed significantly stronger ReHo-reflected activity in the M-Reprocess than in the N-Rest. No region in the STPs-network showed significantly lower ReHo-reflected activity in the M-Reprocess than in the N-Rest.

**Figure 3:**
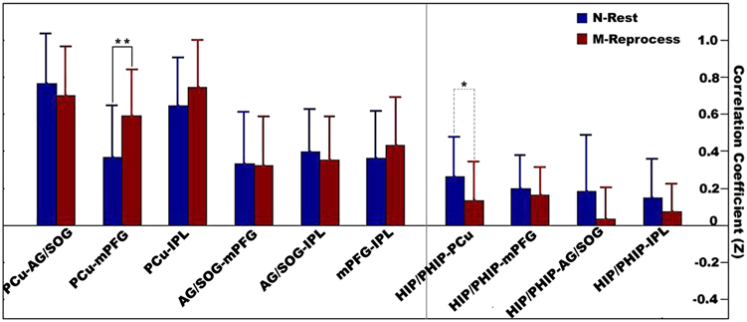
The functional connectivity (FC) between the STPs-network regions. The FC between the PCu and the mPFG was significantly stronger (** *P*<0.05, bonferroni corrected) during the M-Reprocess than during the N-Rest. However, the FC between the HIP/PHIP and the PCu showed a tendency to be weaker (* *P*<0.05, uncorrected) during the M-Reprocess than during the N-Rest.

In addition, we investigated whether offline memory reprocessing could modulate the interactions between the STPs-network regions. As shown in Figure 3, the functional connectivities associated with the HIP/PHIP were significantly weaker than other functional connectivities, both in the N-Rest (*P*<10^−9^) and in M-Reprocess (*P*<10^−10^). In addition, the functional connectivity between the PCu and the mPFG was significantly stronger (*P*<0.005) during the M-Reprocess than during the N-Rest. The functional connectivity between the HIP/PHIP and the PCu was significantly stronger (P<0.05) during the N-Rest than during the M-Reprocess. Other functional connectivities were not significantly different between the N-Rest and the M-Reprocess.

## Discussion

In the present study, we used ReHo-reflected activity to measure brain spontaneous activity during the N-Rest and the M-Reprocess. Our results indicate that the STPs-network regions, including the PCu, the AG/SOG, the IPL, the mPFG and the HIP/PHIP showed significant correlations with frequency of the subjects' mind-wandering during the N-Rest. That is, when subjects had more STPs, these regions showed higher ReHo-reflected activity in order to complete such STPs. These STPs-network regions appear to be located in the default network [23], [25], [28]. Therefore, this finding is highly consistent with previous findings that the activity in the default network regions was associated with STPs [8]. We also found that these regions also showed large ReHo-reflected activity in the M-Reprocess. This result is compatible with previous findings that the HIP/PHIP, the mPFG, the IPL and the PCu are associated with offline memory reprocessing [20]–[22], [29], [30] and that the SOG is associated with visual-related memory processes [31]–[33]. We have found that the activities of those regions in the N-rest are positively correlated with the frequency of mind-wandering in the subjects. According to self-reports provided by the subjects immediately after the scanning, all the subjects had less mind-wandering during the second scan than during the first one (details can be seen in Table S1 of Supplementary Materials). If offline memory reprocessing were not related to STPs, a lower ReHo-reflected activity in those STPs-network regions in the M-Reprocess would be expected because of the lower level of STPs. However, we found that the M-Reprocess further increased the amplitudes of the ReHo-reflected activity in STPs-network regions. Taken together, these results suggest that offline memory reprocessing is an important process in STPs. Therefore, more offline memory reprocessing during the M-Reprocess period than during the N-Rest time will trigger a higher ReHo-reflected activity in the STPs-network regions.

It has been suggested that the amplitude of spontaneous fluctuations (ASF) could also partially indicate the level of regional spontaneous neuronal activity [34], [35]. To further clarify the relationship between spontaneous activity in the default network regions and STPs, we analyzed the ASF in these regions using a power spectrum method. For a given voxel, we first transformed the time series into the frequency domain using a fast Fourier transform and obtained its power spectrum. Because the power at a given frequency is proportional to the square of the amplitude of this frequency component in the original time series, we calculated the square root of the power spectrum at each frequency, and then averaged them across the frequency range to get the ASF value. After calculating the ASF values at all voxels, we also scaled the ASF map by dividing by its mean average value over the entire brain. By investigating the averaged ASF in these STP-network regions, we found that the ASFs in the STPs-network regions were also significantly correlated with the frequencies of the subjects' STPs frequencies (Figure S2 in Supplementary materials). This result further indicates that the spontaneous activity in these regions is modulated by STPs. In addition, the averaged power spectra in the low frequency range were stronger in the PCu, the mPFG and the IPL during the M-Reprocess than during the N-Rest, and were similar in the AG/SOG and HIP/PHIP between the N-Rest and the M-Reprocess (Figure S3 in Supplementary materials). These results indicate that the STP-network regions tended to have a larger signal strength when a subject is involved in STPs during the N-Rest, but showed a similar or even larger signal strength during the M-Reprocess state with a lower level of mind-wandering. Therefore, these results further support our hypothesis that offline memory reprocessing plays an important role in STPs.

From the functional connectivity analysis, we found that most of the functional connectivities between the various STPs-network regions were not significantly different between the N-Rest and the M-Reprocess. This result suggests that offline memory reprocessing and STPs can similarly modulate the interactions within the STPs-network. The interactions among the STPs-network regions reflect a functional integration that may facilitate the retrieval and integration of relevant information components [36]. Interestingly, we found that the functional connectivities associated with the HIP/PHIP tended to be weaker than other functional connectivities in both the N-Rest and in the M-Reprocess. This result suggests that different STPs-network regions may contribute specialized functions that are organized into subsystems. In fact, previous studies have revealed that the brain's default network is comprised of at least two distinct, interacting subsystems: One is the medial temporal subsystem associated with the HIP/PHIP, and the other is a subsystem associated with the mPFG (for a review, see [37]). This is consistent with our finding that the HIP/PHIP and other STPs-network regions are not highly correlated with each other. An explanation for such functional separation is that the HIP/PHIP is associated with receiving memory-related information and comparing/combining distinct memory representations [18], whereas the PCu, the IPL, the AG and the mPFG have been suggested as being associated with high-level offline memory processes, such as self-referential thought, or time-sequencing/organizing of recalled information [38]–[41]. Compared with the N-Rest, the functional connectivity between the PCu and the mPFG were significantly stronger during the M-Reprocess, suggesting the strengthened interaction between the two regions. However, the functional connectivity between the HIP/PHIP and the PCu showed a tendency to be weaker (*P*<0.05, uncorrected) during the M-Reprocess than during the N-Rest. Our ReHo analysis has found that both the HIP/PHIP and the PCu showed increased ReHo-reflected activity during the M-Reprocess than the N-Rest (Figure 2). Therefore, these results indicate that although both the HIP/PHIP and the PCu were more involved in the M-Reprocess, their activities showed a tendency to be de-coherent during the higher demanding state, further suggesting their different roles in the offline memory reprocessing. Taken together, the execution of offline memory reprocessing by the STPs-network would be expected to be a combined effect of functional integration and functional separation among its components. Of course, the low statistical significance makes it difficult to give a strong interpretation for this result. Future studies are still needed to give a picture for the working mechanism of the STPs-network in offline memory reprocessing.

It should be noted that our experimental design could not exclude the possibility completely that the results may be influenced by the passage of time. As the M-Reprocess always occurred after the N-Rest for each participant, it is likely that some confounding factors, such as the difference in the anxiety about the scanner environment, were involved in the main effect of the differences between the two states. Future studies are needed to further address this issue. For example, the inclusions of an appropriate ‘control’ state during which the subjects performed a continuous demanding task would provide a helpful reference for our results. In addition, the inclusion of another group with a different experimental design, such as the N-Rest followed by another N-Rest, could also help to resolve this issue. Of course, the results of an independent experiment will offer stronger evidence for the present study.

Overall, the present study provides a new insight into the functional significance of STPs. Mental processes can be evoked by either external stimuli or internal dynamics occurring spontaneously without a specific task. Although these two kinds of processes are different phenomena, they may share similar underlying mechanisms [42]. Our results suggest that offline memory reprocessing and STPs share a similar neural network, and that offline memory reprocessing is a possible function of STPs. From a physiological viewpoint, offline memory reprocessing plays an important role in the waking-state memory consolidation and memory revision which is necessary for the permanent storage of memory [43], [44]. In addition, considering the similarity between the present STPs-network and the core brain system that mediates past and future thinking [45], it is also possible that offline memory reprocessing plays an important role in combining information from the past and the present to generate predictions about the future [45], [46]. Taken together, it seems reasonable to speculate that the brain automatically wanders, if not required by external tasks, for the offline replaying of encoded information to further enable the consolidation or modification of memory [47]. This is consistent with the perspective that memory can be modified during STPs, and thus gives rise to a dynamic behavior dependent on previous experiences, both external and internal [16], [17].

More generally, STPs are believed to constitute a psychological baseline for human brain functions, during which some other intrinsic mental processes, such as mental imagery and introspective evaluative processes, may also be involved [48]–[50]. Offline memory processing also plays a fundamental role in those intrinsic processes [42]. Therefore, the default network of STPs may be the common basis for all those intrinsic mental processes. During a deliberate goal-directed task state, STPs are suspended [1], [8], [23]; but when external demands are low, STPs automatically occur, driven by internal dynamics. In other words, rather than letting time pass with an idle brain, there is a spontaneous switch between externally-driven and internally-driven processes, which highly increases the effectiveness of the brain.

## Materials and Methods

### Subjects

This study was approved by the Human Research Ethics Committee of Xuanwu Hospital of Capital Medical University. Thirteen subjects (7 female, 6 male; mean age 25.3 years, range 23–28 years) participated in the study. Written informed consent was obtained from all subjects. All participants were right-handed [51] and had no history of neurological or psychiatric disorders. Twelve of the thirteen subjects completed a 12-item daydream frequency scale of the Imaginal Process of Inventory [26] to measure each individuals' tendency to mind-wander. Only these twelve subjects were used in the subsequent analysis.

### Data acquisition

All images were scanned on a 3.0 Tesla Siemens MR system. A foam pad and headphones were used to reduce head motion and scanner noise. Blood oxygen level dependent (BOLD) images of the entire brain were acquired in 32 axial slices by using an echo-planar imaging sequence [TR/TE = 2000/30 ms, flip angle (FA) = 90°, field of view (FOV) = 22 cm, matrix = 64×64, thickness = 3 mm, gap = 1 mm].

In the present study, participants were first instructed to maintain a natural resting state (N-Rest) during a six-minute fMRI scan by closing their eyes, relaxing their minds and stopping any structured mental processes such as counting. More importantly, all participants were asked to stay awake during the examinations. The scanning lasted for 6 minutes and 180 BOLD images were acquired.

About one hour after the first scan (mean: 1 hour, range: 45–75 minutes), each subject was asked to memorize a landscape picture for about 10 minutes. The picture was selected from the International Affective Picture System (IAPS). Immediately after this procedure, the subject was scanned a second time using the same parameters as above. During the second scan, subjects were instructed to close their eyes and recall the content of the picture as much as possible (offline memory reprocessing state, M-Reprocess). This M-Reprocess scanning procedure also lasted for 6 minutes and 180 BOLD images were acquired.

We made the experiment design based on the following considerations: (1) the N-Rest is a task-free state, and the effect of STPs is easy to be identified; (2) we hypothesized that the offline memory reprocessing is an important function of STPs, and the M-Reprocess mainly involve the offline memory reprocessing. According to our pre-experiment, if the M-reprocess was taken before the N-Rest, the subjects could not control retrieving of the memorized information during the N-Rest. In this situation, it will be difficult to discriminate the effect of natural STPs from the goal-directed memory reprocessing processes during the N-Rest. However, the N-Rest is a natural resting state, and the subjects reported no particular influence on the latter M-Reprocess state.

### Data preprocessing

The same preprocessing procedures were used for both the scanning datasets. Most of the steps were carried out using statistical parametric mapping (SPM2, http://www.fil.ion.ucl.ac.uk/spm/). Because of the instability of the initial signal and the subjects' adaptation to the situation, the first 10 images were discarded. The remaining images were first corrected for within-scan acquisition time differences between slices and then realigned to the first volume to correct for inter-scan head motions. Next, we spatially normalized the realigned images to the standard EPI template and re-sampled them to a voxel size of 3×3×3 mm^3^. Subsequently, we used a multiple regression procedure to remove other possible resources of artifacts [28]: (1) six motion parameters, (2) linear drift, and (3) the drift of whole brain signals averaged over the entire brain.

### Regional homogeneity (ReHo) Analysis

For a given voxel, the ReHo approach calculates the Kendall's coefficient of concordance (KCC) [52] of the time series of this voxel with those of its nearest neighbors:
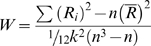
(1)where *W* ranges from 0 to 1; 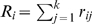, where 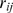 is the rank of the *i-th* time point in the *j-th* voxel; 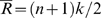 is the mean of the 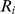; *n* is the number of time points in each voxel time series (here *n* = 170); and *k* is the number of time series within the measured cluster (here *k* = 27, the central voxel plus its 26 neighbors). An individual *W* map (i.e. ReHo map) was obtained on a voxel-by-voxel basis using the data from each subject.

In the present study, we first calculated the ReHo-reflected activity map on a voxel-by-voxel basis for each subject, in both the N-Rest and the M-Reprocess. Then we standardized each ReHo map by dividing by its mean average value over the entire brain. This procedure is similar to that used in PET studies [23] and provides standard ReHo maps with a mean value of 1.

### Functional connectivity Analysis

Recently, fMRI studies have demonstrated that spontaneous BOLD fluctuations are coherent within specific neuro-anatomical systems [25], [28], [34], [53]–[59]. Based on the conventional functional connectivity methodology [34], we calculated the functional connectivity among the above acquired STPs-network regions in both the N-Rest and the M-Reprocess to evaluate their interactions with each other as follows: (1) the reference time series of each region was calculated by averaging the time series across all the voxels in that region; (2) Pearson's correlation coefficients were calculated between each pair of reference time series; (3) Fisher's *r*-to-*z* transformation was applied to improve the normality of these correlation coefficients. To investigate whether offline memory reprocessing could modulate the interactions within the STPs-network, these *z* values were then entered into a random-effect paired-*t* test to determine the functional connectivities that were significantly different between the N-Rest and the M-Reprocess.

## Supporting Information

Figure S1 Brain regions whose ReHo-reflected activity was significantly correlated with subjects' daydreaming/mind-wandering frequencies (total score = 60) during the N-Rest (P<0.05, t>1.82 for individual voxels; and cluster size >5 voxels). It can be seen that the core regions associated with the brain's default network, including the ventral and dorsal medial prefrontal gyrus (mPFG), the posterior cingulate cortex (PCC), the precuneus (PCu), the inferior parietal lobule (IPL), the angular gyrus (AG), the superior occipital gyrus (SOG), the lateral temporal cortex (LTC), and the hippocampus/parahippocampus (HIP/PHIP), showed significant correlations with the subjects' spontaneous thought processes.
(4.72 MB TIF)

Figure S2 The relationship between the amplitude of spontaneous signal fluctuations (ASF) in the STPs-network regions and subjects' daydreaming/mind-wandering frequencies. (A) PCu. (B) AG/SOG. (C) mPFG. (D) HIP/PHIP. (E) IPL.
(6.43 MB TIF)

Figure S3 Mean power spectra of the STP-network regions in the N-Rest and the M-Reprocess The power spectra in the PCu, the mPFG and the IPL throughout the low frequency portion (0.01–0.08 Hz, we show this frequency range because it holds most of the signal power) were stronger during the M-Reprocess than during the N-Rest. And the averaged Fourier power spectra in the AG/SOG and the HIP/PHIP were not significantly different between the N-Rest and the M-Reprocess.
(9.00 MB TIF)

Table S1 (0.04 MB DOC)
