# Supplementary figures and images for: Offline Memory Reprocessing: Involvement of the Brain's Default Network in Spontaneous Thought Processes
Source: PLoS One. 2009 Mar 17;4(3):e4867. doi: 10.1371/journal.pone.0004867 (PMC2653727; doi:10.1371/journal.pone.0004867)

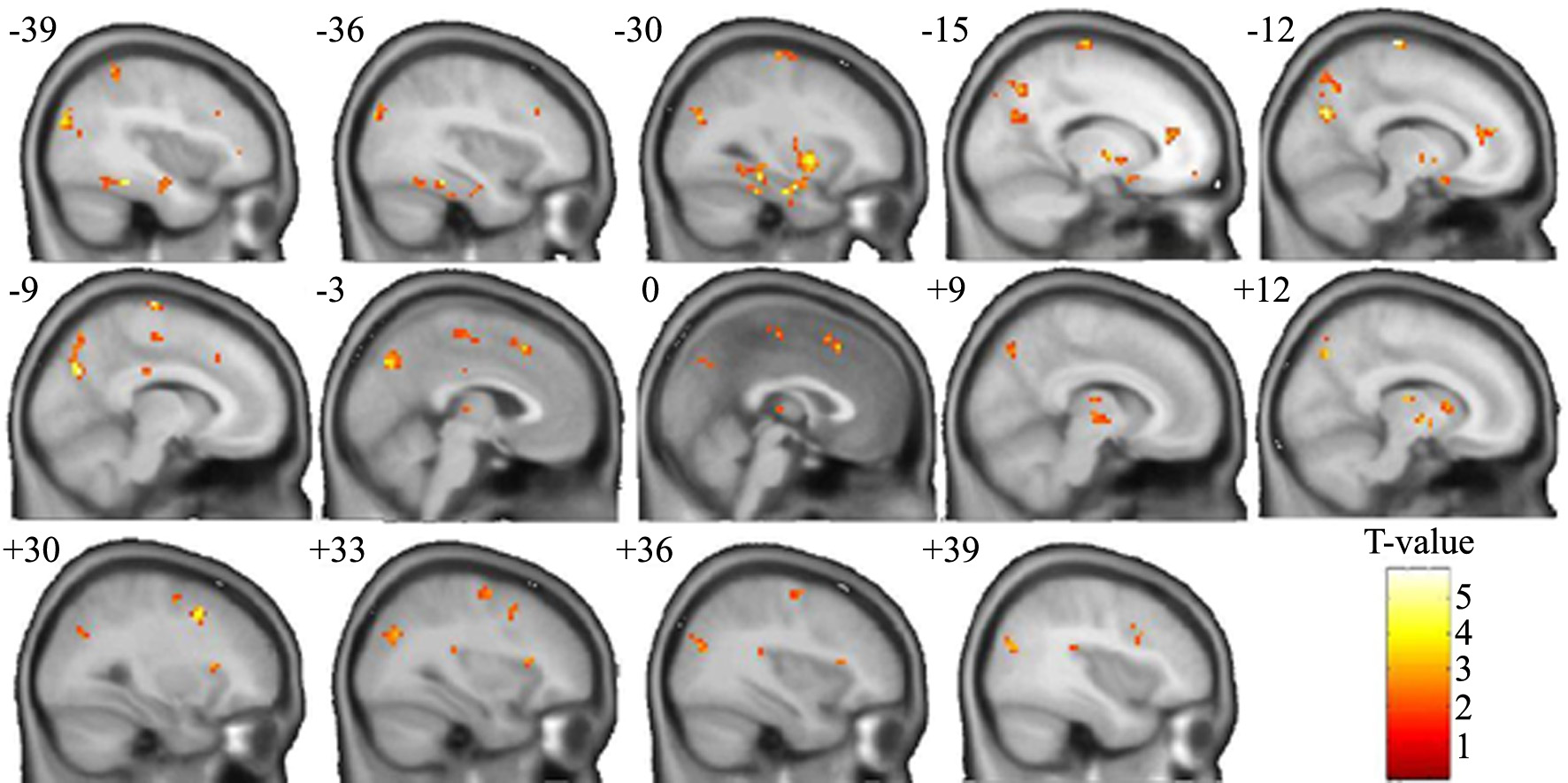

Supplement: Figure S1 — Brain regions whose ReHo-reflected activity was significantly correlated with subjects' daydreaming/mind-wandering frequencies (total score = 60) during the N-Rest (P<0.05, t>1.82 for individual voxels; and cluster size >5 voxels). It can be seen that the core regions associated with the brain's default network, including the ventral and dorsal medial prefrontal gyrus (mPFG), the posterior cingulate cortex (PCC), the precuneus (PCu), the inferior parietal lobule (IPL), the angular gyrus (AG), the superior occipital gyrus (SOG), the lateral temporal cortex (LTC), and the hippocampus/parahippocampus (HIP/PHIP), showed significant correlations with the subjects' spontaneous thought processes. (4.72 MB TIF) [file pone.0004867.s001.tif]

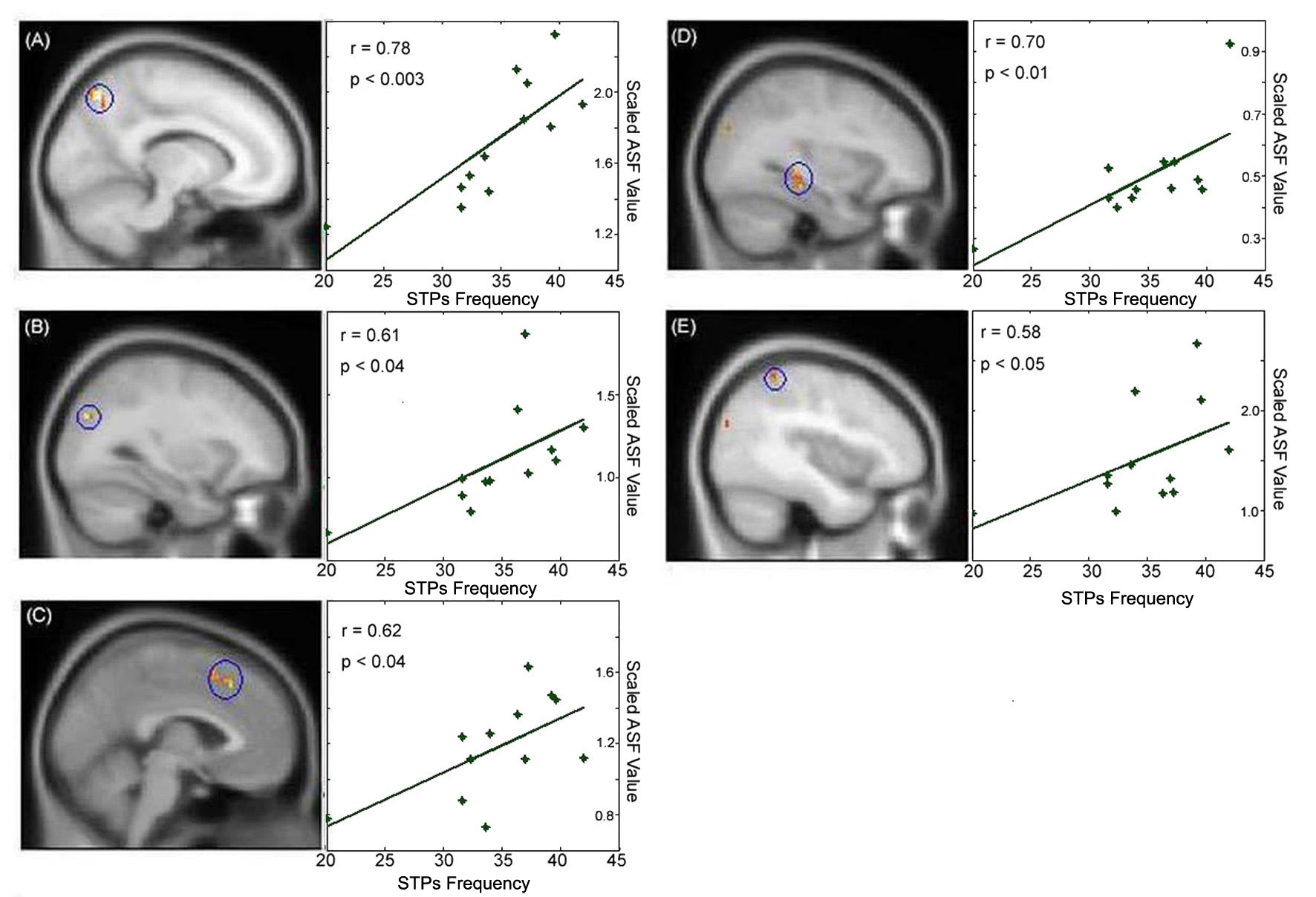

Supplement: Figure S2 — The relationship between the amplitude of spontaneous signal fluctuations (ASF) in the STPs-network regions and subjects' daydreaming/mind-wandering frequencies. (A) PCu. (B) AG/SOG. (C) mPFG. (D) HIP/PHIP. (E) IPL. (6.43 MB TIF) [file pone.0004867.s002.tif]

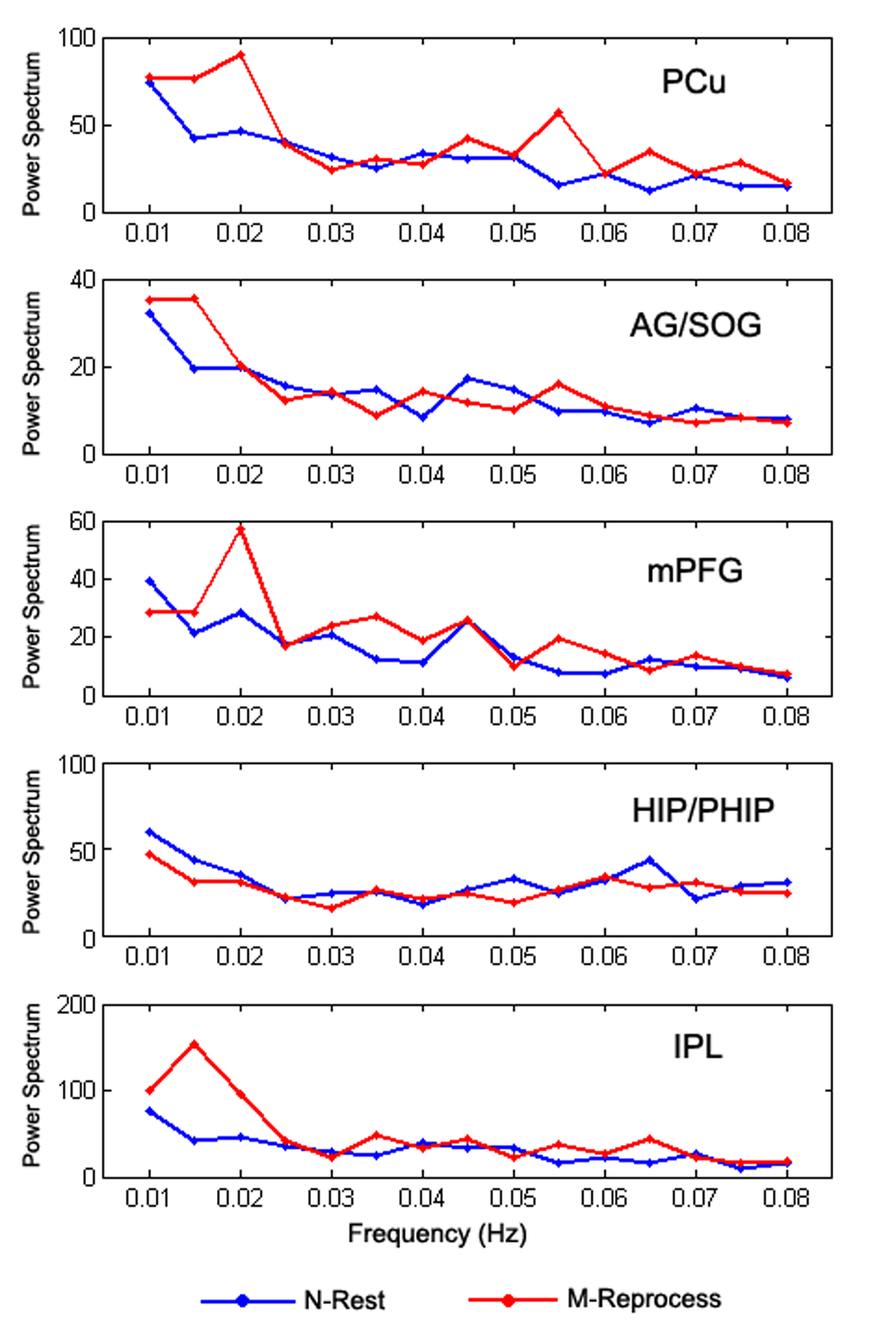

Supplement: Figure S3 — Mean power spectra of the STP-network regions in the N-Rest and the M-Reprocess The power spectra in the PCu, the mPFG and the IPL throughout the low frequency portion (0.01–0.08 Hz, we show this frequency range because it holds most of the signal power) were stronger during the M-Reprocess than during the N-Rest. And the averaged Fourier power spectra in the AG/SOG and the HIP/PHIP were not significantly different between the N-Rest and the M-Reprocess. (9.00 MB TIF) [file pone.0004867.s003.tif]
