# Supplementary material for: Offline Memory Reprocessing: Involvement of the Brain's Default Network in Spontaneous Thought Processes
Source: PLoS One. 2009 Mar 17;4(3):e4867. doi: 10.1371/journal.pone.0004867 (PMC2653727; doi:10.1371/journal.pone.0004867)
Supplement: Table S1 — (0.04 MB DOC) [file pone.0004867.s004.doc]

**Table S1: The STPs frequency scale measured by Imaginal Process of Inventory (IPI), and self-reported STPs proportions during the N-Rest and the M-Reprocess.**

| **Subjects** | **IPI-measured STPs Frequency Scale** | **Self-reported STPs Proportion** | |
| --- | --- | --- | --- |
|  |  | **N-Rest** | **M-Reprocess** |
| **S001** | 37 | 40% | 20% |
| **S002** | 32 | 20% | 10% |
| **S003** | 40 | 25% | 10% |
| **S004** | 34 | 35% | 10% |
| **S005** | 37 | 15% | 10% |
| **S006** | 34 | 40% | 20% |
| **S007** | 32 | 40% | 5% |
| **S008** | 36 | 50% | 40% |
| **S009** | 32 | 20% | 10% |
| **S010** | 42 | 55% | 30% |
| **S011** | 39 | 45% | 15% |
| **S012** | 20 | 15% | 10% |

It should be noted that the self-reported proportions were a little arbitrary. We just use these data to indicate the existence of STPs and to descriptively investigate the proportion differences between the N-Rest and the M-Reprocess (N-Rest > M-Reprocess, *P* < 0.0001, paired-*t* test). The IPI-measured STPs frequency and the self-reported STPs proportions during the N-Rest showed a nearly significant correlation (r=0.56, *P* < 0.06), suggesting that individuals exhibit stable differences in their propensity to have STPs in the natural state. There was no significant correlation between the IPI-measured STPs frequency and the self-reported STPs proportions during the M-Reprocess (r=0.39, *P* < 0.2), suggesting that the estimation of STPs may be influenced by the goal-directed processing during the M-Reprocess.
